# Supplementary material for: Oxy210 Inhibits Hepatic Expression of Senescence-Associated, Pro-Fibrotic, and Pro-Inflammatory Genes in Mice During Development of MASH and in Hepatocytes In Vitro
Source: Cells. 2025 Aug 2;14(15):1191. doi: 10.3390/cells14151191 (PMC12346697; doi:10.3390/cells14151191)
Supplement: Supplementary file 1 [file cells-14-01191-s001.zip › cells-3761193-supplementary.pdf]

Supplementary Table S1

Primers used for mouse genes

Rpl4	5′- GGAAGTTGGATGAGCTGTAT-3′<br>5′- TCAAGATTCTGCTAAGGTCG-3′
Tnf-a	5’-CAGGCGGTGCCTATGTCTC-3’<br>5’-CGATCACCCCGAAGTTCAGTAG-3’
Il-1b	5’-GCAACTGTTCCTGAACTCAACT-3’<br>5’-ATCTTTTGGGGTCCGTCAACT-3’
Tgfb1	5’-CTCCCGTGGCTTCTAGTGC-3’<br>5’-GCCTTAGTTTGGACAGGATCTG-3’
Ctgf	5’-GGGCCTCTTCTGCGATTTC-3’<br>5’-ATCCAGGCAAGTGCATTGGTA-3’
Ccl2	5’-TTAAAAACCTGGATCGGAACCAA-3’<br>5’-GCATTAGCTTCAGATTTACGGGT-3’
Il-6	5’-TAGTCCTTCCTACCCCAATTTCC-3’<br>5’-TTGGTCCTTAGCCACTCCTTC-3’
Gli1	5’-GAAGCCGAGCCGAGTATC-3’<br>5’-GGTGAGTAGACAG AGGTTGG-3’
Acta2	5’-GTCCCAGACATCAGGGAGTAA-3’<br>5’-TCGGATACTTCAGCGTCAGGA-3’
Col1a1	5’- GCTCCTCTTAGGGGCCACT-3’<br>5’- CCACGTCTCACCATTGGGG-3’
p16	5’- CGCAGGTTCTTGGTCACTGT-3’<br>5’- TGTTCACGAAAGCCAGAGCG-3’

To be continued

Ptch1	5’-CCATCGGCGACAAGAACC-3’<br>5’-CCAGCACAGCAAAGAAATACC-3’
Spp1	5’- CCATCTCAGAAGCAGAATCTCC-3’<br>5’- ATCGTCATCATCGTCGTCC-3’
p21	5’-CCTGGTGATGTCCGACCTG-3’<br>5’-CCATGAGCGCATCGCAATC-3’
p15	5’-CCCTGCCACCCTTACCAGA-3’<br>5’-CAGATACCTCGCAATGTCACG-3’
p53	5’-CTCTCCCCCGCAAAAGAAAAA-3’<br>5’-CGGAACATCTCGAAGCGTTTA-3’
Pai-1	5’-TTCAGCCCTTGCTTGCCTC-3’<br>5’-ACACTTTTACTCCGAAGTCGGT-3’
Chop	5’-CTGGAAGCCTGGTATGAGGAT-3’<br>5’-CAGGGTCAAGAGTAGTGAAGGT-3’
Nox2	5’-AGTGCGTGTTGCTCGACAA-3’<br>5’-GCGGTGTGCAGTGCTATCAT-3’

Primers used for human genes

PSMB4	5′- TCGGCCAGATGGTGATTGAT-3′<br>5′- CAGCATAGCCTCCGATGACC-3′
P15	5′- ACGGAGTCAACCGTTTCGGGAG -3′<br>5′- GGTCGGGTGAGAGTGGCAGG -3’
P21	5′- TGTCCGTCAGAACCCATGC -3’<br>5′- AAAGTCGAAGTTCCATCGCTC -3’
TGFB1	5′- CAATTCCTGGCGATACCTCAG -3’<br>5′- GCACAACTCCGGTGACATCAA -3′
TNFA	5′-GAGGCCAAGCCCTGGTATG-3′<br>5′-CGGGCCGATTGATCTCAGC-3′
