# Supplementary figures and images for: Oxy210 Inhibits Hepatic Expression of Senescence-Associated, Pro-Fibrotic, and Pro-Inflammatory Genes in Mice During Development of MASH and in Hepatocytes In Vitro
Source: Cells. 2025 Aug 2;14(15):1191. doi: 10.3390/cells14151191 (PMC12346697; doi:10.3390/cells14151191)

## Slide 1
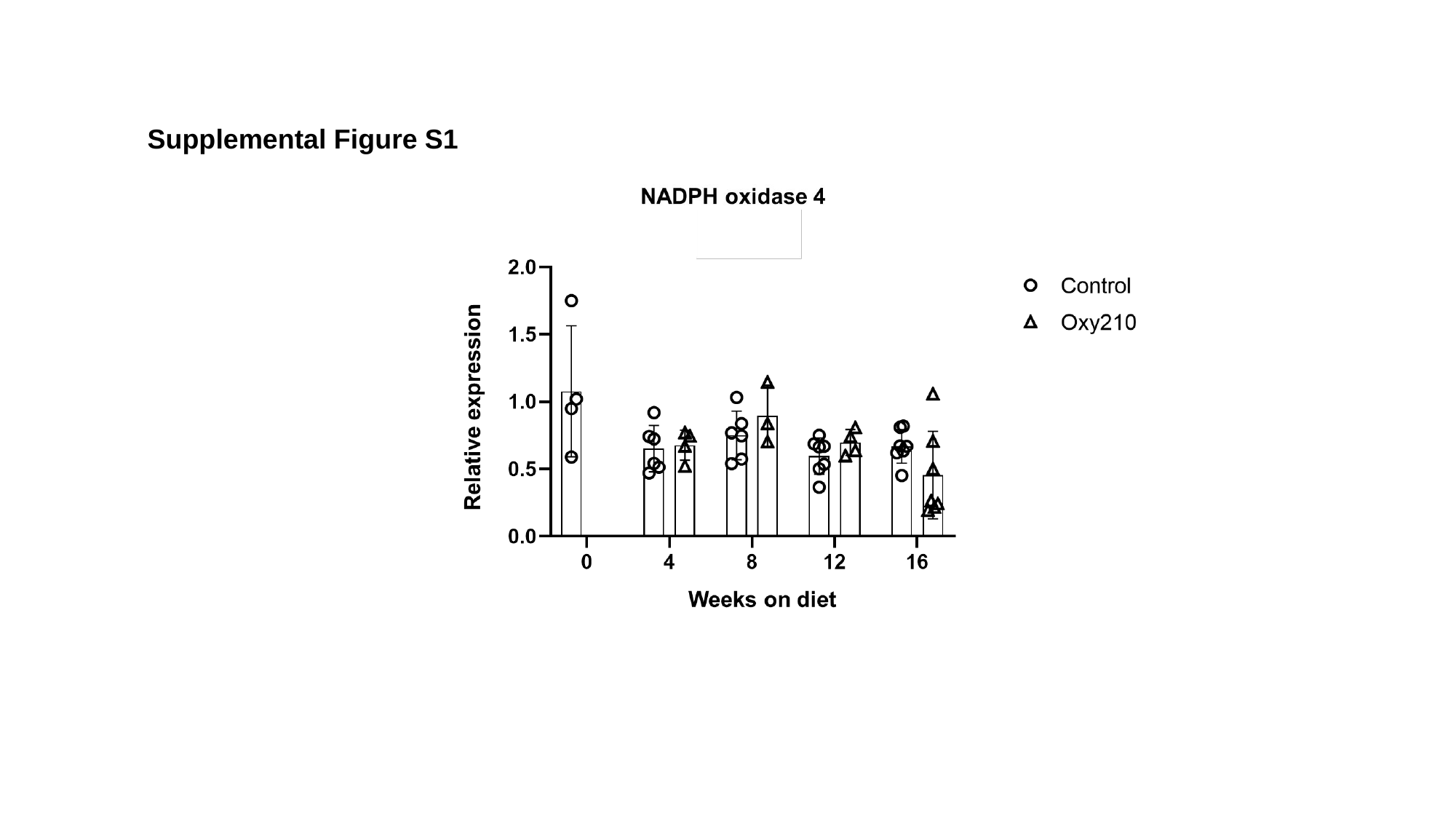

Supplemental Figure S1

Supplement: Supplementary file 1 [file cells-14-01191-s001.zip › cells-3761193-supplementary.pptx]
